# Molecular Insights in Atrial Fibrillation Pathogenesis and Therapeutics: A Narrative Review

**DOI:** 10.3390/diagnostics11091584

**Published:** 2021-08-31

**Authors:** Konstantinos A. Papathanasiou, Sotiria G. Giotaki, Dimitrios A. Vrachatis, Gerasimos Siasos, Vaia Lambadiari, Konstantinos E. Iliodromitis, Charalampos Kossyvakis, Andreas Kaoukis, Konstantinos Raisakis, Gerasimos Deftereos, Theodore G. Papaioannou, Georgios Giannopoulos, Dimitrios Avramides, Spyridon G. Deftereos

**Affiliations:** 1 Medical School, National and Kapodistrian University of Athens, 11527 Athens, Greece; kpapathanasiou91@gmail.com (K.A.P.); sotiria.giotaki@yahoo.com (S.G.G.); dvrachatis@gmail.com (D.A.V.); ger_sias@hotmail.com (G.S.); vlambad@otenet.gr (V.L.); teogpap@gmail.com (T.G.P.); 2 Evangelisches Krankenhaus Hagen-Haspe, Clinic for Cardiology and Electrophysiology, 58135 Hagen, Germany; konstantinos.iliodromitis@gmail.com; 3 Department of Cardiology, “G. Gennimatas” General Hospital of Athens, 11527 Athens, Greece; ckossyvakis@gmail.com (C.K.); andreaskaoukis@yahoo.gr (A.K.); kraisakis@yahoo.co.uk (K.R.); gerasimosd@gmail.com (G.D.); d_avramides@yahoo.com (D.A.); 4 Medical School, Aristotle University of Thessaloniki, 54124 Thessaloniki, Greece; ggiann@med.uoa.gr

**Keywords:** atrial fibrillation pathophysiology, rotors and focal impulses, fibrosis, extracellular matrix, fibroblasts, calcium homeostasis, inwardly rectifying potassium current, connexins, epicardial adipose tissue, autonomic nervous system, inflammasome, immune cells, chemokines, oxidative stress, ATP depletion, mitochondrial oxidative phosphorylation, derailed proteostasis, adenosine monophosphate-regulated protein kinase, therapeutic implications

## Abstract

The prevalence of atrial fibrillation (AF) is bound to increase globally in the following years, affecting the quality of life of millions of people, increasing mortality and morbidity, and beleaguering health care systems. Increasingly effective therapeutic options against AF are the constantly evolving electroanatomic substrate mapping systems of the left atrium (LA) and ablation catheter technologies. Yet, a prerequisite for better long-term success rates is the understanding of AF pathogenesis and maintenance. LA electrical and anatomical remodeling remains in the epicenter of current research for novel diagnostic and treatment modalities. On a molecular level, electrical remodeling lies on impaired calcium handling, enhanced inwardly rectifying potassium currents, and gap junction perturbations. In addition, a wide array of profibrotic stimuli activates fibroblast to an increased extracellular matrix turnover via various intermediaries. Concomitant dysregulation of the autonomic nervous system and the humoral function of increased epicardial adipose tissue (EAT) are established mediators in the pathophysiology of AF. Local atrial lymphomononuclear cells infiltrate and increased inflammasome activity accelerate and perpetuate arrhythmia substrate. Finally, impaired intracellular protein metabolism, excessive oxidative stress, and mitochondrial dysfunction deplete atrial cardiomyocyte ATP and promote arrhythmogenesis. These overlapping cellular and molecular alterations hinder us from distinguishing the cause from the effect in AF pathogenesis. Yet, a plethora of therapeutic modalities target these molecular perturbations and hold promise in combating the AF burden. Namely, atrial selective ion channel inhibitors, AF gene therapy, anti-fibrotic agents, AF drug repurposing, immunomodulators, and indirect cardiac neuromodulation are discussed here.

## 1. Introduction

Cardiac senescence, largely attributed to aging, hypertension, obesity, as well as genetic predisposition, has been associated with atrial fibrillation (AF) genesis and progression [1]. AF is expected to affect 6–16 million individuals in the United States, 14 million in Europe, and 72 million in Asia by 2050 [2], imposing a surge with economic and social implications for the public health care systems.

One out of three patients with AF will develop heart failure, and 20–30% of ischemic strokes are attributed to AF, increasing morbidity and mortality [3].

Almost four centuries have elapsed since 1628 when William Harvey was probably the first to describe AF in animals [4]. Nowadays, different ablation strategies have revolutionized AF treatment. However, a ‘’ceiling’’ for more durable long-term success (strictly defined as no occurrence of AF) seems to be reached. Success rates range between 65 and 78%, requiring an in-depth understanding of mechanistic links [5].

This narrative review aims to summarize fundamental aspects pertaining to AF pathophysiology in humans. Therapeutic implications are also discussed.

## 2. Atrial Fibrillation Pathogenesis

### 2.1. Mechanistic Approach

Increased focal atrial triggered activity, mainly due to delayed afterdepolarizations (DADs) and micro-reentrant circuits are the main electrophysiological mechanisms in all types of AF (paroxysmal, persistent, and permanent) [6].

In 1998, Haïssaguerre M et al. suggested that pulmonary vein (PV) ectopic activity is implicated in AF pathogenesis, paving the way for pivotal ablative therapeutic modalities such as pulmonary vein isolation (PVI) [7]. Abnormal atrial repolarization (exaggerated beat-to-beat oscillations in action potential duration [APD]) and decreased atrial conduction are shown to mitigate re-entry in patients with AF [8,9].

A frequency-domain approach, utilized to explain AF initiation and maintenance, underscores that AF mechanistic links might be less chaotic than originally thought. In particular, focal impulse and rotor modulation (FIRM) mapping has already achieved a ‘’panoramic’’ bi-atrial view and suggests that a small number of stable high-frequency re-entrant sources (rotors) perpetuate AF fibrillatory waves. This spatiotemporally ordered AF substrate was successfully targeted via FIRM-guided ablation with salutary long-term effects in CONFIRM and RADAR AF trials [10,11]. Yet, two recent meta-analyses report discordant results regarding the benefit of a combined AF rotors ablation and PVI as opposed to PVI alone [12,13].

### 2.2. Molecular Pathophysiology

#### 2.2.1. Ionic Perturbations

The study of electric remodeling in human atria focuses, mainly, on altered calcium kinetics, impaired inwardly rectifying potassium currents, and gap junction changes.

Abnormal intracellular calcium (Ca^2+^) handling is critical in triggering DADs and thus increased atrial ectopic activity. In human AF models, enhanced spontaneous sarcoplasmic reticulum (SR) Ca^2+^ release has been attributed to ryanodine receptor (RyR2) dysregulation [14,15], Ca^2+^/calmodulin-dependent protein kinase-II (CaMKII) hyperactivity [16,17,18], or SPEG (striated muscle preferentially expressed protein kinase), a regulator of RyR2 phosphorylation and downregulation [19].

L-type Ca [2] current (I_Ca,L_) attenuation leads to atrial APD shortening and seems to be implicated in AF maintenance [20,21]. An effect is at least partially driven by microRNA-21 and microRNA-328 in humans [22,23]. Early re-activation of I_Ca,L_ current can also lead to early afterdepolarizations (EADs) and AF initiation [24]. Interestingly, impaired calcium homeostasis in human cardiomyocytes leads to inositol-trisphosphate-receptor (IP3R)/CAMKII signaling, which in turn decrease I_Ca,L_ density [25].

Inwardly rectifying potassium (Kir) current (I_K1_) as well as acetylcholine-activated potassium current (I_K,Ach_) are enhanced in AF patients and shorten atrial APD [26,27]. MicroRNA-26 and microRNA-1 downregulation leads to increased Kir2.1 protein expression and establishes atrial re-entry via I_K1_ current activation [28,29]. Both I_K1_ and I_K,Ach_ currents are critically involved in maintaining a left-to-right dominant frequency gradient in paroxysmal AF (PAF) subjects and explain AF drivers formation (rotors and focal impulses) [30].

Cardiac connexins create gap junctions, facilitating cell-to-cell electrical and molecular signaling [31]. Ultra-structural changes of atrial connexins are noticed in human AF experiments. Connexin-43 (Cx43) dysregulation is present in the atria of AF patients [32] and seems to be regulated through interplay between microRNA-613 and long noncoding RNA HOTAIR (HOX transcript antisense RNA) [33]. Connexin-40 (Cx40) is mainly found in the atrial myocardium and is associated with AF development, as shown in various genetic analyses [34,35,36]. Increased lateralization and hyperphosphorylation of either Cx43 or Cx40 are implicated in human AF pathophysiology [37,38,39].

#### 2.2.2. Structural Changes

Atrial fibrosis is the result of increased fibroblast activity with heterogeneous patchy areas of collagen type I depositions. This favors longer AF periods, LA enlargement, distortion of intercellular electrical coupling, and perpetuation of AF fibrillatory waves [40].

Of note, delicate 3D human atria models suggested that fibrosis reduces atrial conduction velocity and stabilizes rotors and re-entrant circuits [41]. A study including patients with persistent AF, who underwent LA tissue characterization with MRI scans and concomitant high-density mapping of the LA, demonstrated increased AF rotor activity in areas of relatively low and patchy late-gadolinium enhancement (LGE) [42]. Bioinformatics’ analysis has recently revealed that LA–PV junction demonstrates distinct gene expression differences in AF patients as compared to sinus rhythm (SR) controls, favoring extracellular matrix (ECM) synthesis and chemokine up-regulation [43].

TGF-β signaling is strongly associated with these structural changes [44]. Various microRNAs and long noncoding RNAs seem to regulate cardiac fibroblast profibrogenic activity [45,46,47,48,49]. A recently discovered crosstalk between Slit2-Robo1 and TGF-β1/Smad pathways promises potential therapeutic targets against atrial fibrosis [50].

Furthermore, platelet-derived TGF-β secretion stimulates fibroblast proliferation, setting a vicious cycle of atrial fibrosis [51]. Platelet-derived growth factor (PDGF) also leads to increased cardiac fibroblast activity [52] and eventually to atrial fibrosis [53].

Mitogen-activated protein kinase 1 (MAPK1) overexpression, evident in cardiac fibroblast from AF patients, stimulates collagen deposition. An effect that can be mitigated by microRNA-450a-2-3p [54].

Angiotensin II (Ang II) is also known to induce critical ECM changes (increased collagen deposition and metalloproteinase activity) via JAK/STAT3 molecular pathway [55]. Ang II receptor type 1 (AT1) is up-regulated in the LA of subjects suffering AF [56]. Inhibition of AT1 restores intracellular calcium homeostasis and prevents arrhythmogenesis [57]. In addition, fibroblast growth factor 23 (FGF23) is involved in atrial fibrogenesis via increased oxidative stress and STAT3/SMAD3 signaling [58].

Connective tissue growth factor (CTGF) levels in human atrial fibroblasts and epicardial adipose tissue (EAT) are positively correlated with atrial fibrosis and AF arrhythmogenesis [59,60]. MicroRNA-132 and Ang II regulate CTGF levels in human atria [61,62].

Finally, atrial tissue calcitonin levels are inversely correlated with atrial arrhythmogenesis. A recent study suggested that calcitonin halts cardiac fibroblast overactivity and prevents ECM turnover [63].

Apart from generating a fibrotic substrate, cardiac fibroblasts affect cell-to-cell electrical coupling and exhibit altered electrophysiological properties in humans suffering from AF compared to SR controls [64]. This finding necessitates further evaluation, especially in the light of mechanistic data suggesting that fibroblast proliferation leads to complex fractionated atrial electrograms (CFAEs) genesis [65] and action potential propagation block in pulmonary veins [66].

#### 2.2.3. Epicardial Adipose Tissue (EAT) and Autonomic Nervous System (ANS)

From a mechanistic point of view, the EAT in patients with persistent AF (PeAF) seems to be related to rotors capable of maintaining AF [67] and positively associated with low voltage areas, reduced conduction velocity, and CFAE [68,69].

EAT is a known cause of electrophysiological changes, such as heterogeneous atrial conduction slowing. These alterations have been attributed to Cx40 lateralization, excessive fibrosis, and heterogeneous adipose infiltration of the affected atria [70].

Interestingly, a unique molecular footprint has recently been shown in EAT from AF subjects. In particular, EAT derived extracellular vesicles (EVs) exert profibrotic/proinflammatory effects on the neighboring atrial tissue, promoting arrhythmogenesis [71]. In addition, EAT expansion seems to be positively regulated through increased atrial natriuretic peptide (ANP) levels [72].

EAT is metabolically active, and paracrine secretion of inflammatory mediators (IL-1β, among others) [73,74] is associated with atrial fibrillation in humans [75].

EAT-mediated atrial fibrosis has been linked to the PeAF subtype, and CD8^+^ lymphocyte infiltrates are seemingly involved [76]. Angiopoietin-like protein 2 (Angptl2), YKL-40, CTGF, activin A (TGF-β superfamily) upregulation and Omentin-1 downregulation in human EAT are also implicated in atrial fibrosis and AF development [60,77,78,79,80].

Intrinsic cardiac ANS is organized in a network of ganglionated plexi (GP), which are accommodated in EAT, mainly around PVs [81].

Various methods of cardiac ANS assessment (heart rate variability, [82,83] skin sympathetic nerve activity [84], metaiodobenzylguanidine (MIBG) scintigraphy [85]) imply that ANS instability is implicated in human AF pathogenesis.

The clinical impact of ANS modulation (GP ablation in addition to PVI in patients with PAF [86], chemical; botulinum toxin [87,88] or calcium chloride [89]; autonomic denervation in cardiac surgery patients, transcutaneous vagal nerve stimulation [90]) in managing AF further strengthens the role of autonomic remodeling in AF pathophysiology, and it is further discussed in Section 3: Therapeutic Perspectives.

#### 2.2.4. The Role of Inflammation

Local inflammation is apparent in AF pathophysiology since human LA tissue examination has revealed an infiltrate of varying immune cells (neutrophils, proinflammatory CD68^+^ macrophages, CD8^+^ and CD3^+^ lymphocytes) in AF subjects [91,92,93,94].

IL-6 secretion seems to be a critical mediator in suppressing regulatory T cell function and triggering atrial fibrosis [95]. In addition, macrophage migration inhibitory factor (MIF) release, an early mediator in inflammation cascade, was previously shown to suppress I_(Ca,L)_ current and is also associated with AF genesis [96].

Serum levels of Interleukin-2 soluble receptor and TNF-α soluble receptor are among the stronger predictors of new-onset AF, as assessed via machine learning algorithms in Multi-Ethnic Study of Atherosclerosis (MESA) [97]. This observation is in accordance with other indices of systematic inflammation (TNF-α, hs-CRP, IL-6, IL-8, and IL-18) [98,99,100,101], all of which are up-regulated in the serum of patients with AF. These findings suggest the interplay of an atrial-specific and systematic hyperinflammatory state in AF subjects. Additionally, elevated baseline hs-CRP levels independently predicted arrhythmia recurrence post-ablation and are positively associated with low LA voltage areas, rotors, and non-PV ectopic foci [102].

NLRP3 (NACHT, LRR, and PYD domain-containing protein 3) inflammasome activity is also enhanced in atrial cardiomyocytes from AF patients and brings about electroanatomic remodeling [103]. Post-operative AF (POAF) patients were shown to express a higher level of the activated inflammasome in their atrial tissue, an observation linked to enhanced spontaneous SR Ca^2+^ release and DADs formation [104].

From a clinical perspective, recent evidence suggests that immunomodulatory agents, such as corticosteroids and colchicine, have a preventive role in POAF development [105,106,107,108] and support the fundamental role of inflammatory pathways in managing AF.

#### 2.2.5. The Role of Proteostasis, Oxidative Stress, and Mitochondrial Bioenergetics

The role of metabolic stress is increasingly recognized in AF pathophysiology, and it is discussed below in view of impaired protein cycling, oxidative stress, and mitochondrial dysfunction.

Proteostasis is defined as the balance between protein synthesis, folding, and degradation [109]. Impaired protein homeostasis is observed in human cellular aging as well as in cardiac diseases [110]. Derailed proteostasis exhibited through heat shock proteins (HSPs) up-regulation, calpain hyperactivity, and autophagosome formation is involved in AF genesis.

HSPs are produced as a response to cellular stress and stabilize other intracellular proteins. HSP27 was previously shown to be up-regulated in the atria of PAF patients and attenuates stress-induced structural changes (myolysis) [111]. In addition, low baseline HSP27 is associated with low LA voltage areas, non-PV foci, and decreased arrhythmia free intervals in patients undergoing ablation for PAF [112]. A more recent study suggests that post-ablation rise in serum HSP27 levels are predictive of arrhythmia recurrence, while baseline levels of different HSPs are of no clinical significance, thus creating a need for further research [113].

Atrial tissue from PeAF patients demonstrates increased macroautophagy (a process of autophagosome formation and eventually lysosomal degradation of damaged proteins), which is linked to reduced I_(Ca,L)_ current and atrial APD shortening in animal studies [114].

Calpain I (a non-lysosomal proteolytic enzyme) activity is enhanced in atrial myocytes of both PAF and PeAF patients and has been linked with APD shortening [115]. Histone deacetylase 6 (HDAC6) hyperactivity, evident in human AF atria, disrupts cytoskeleton (microtubules) and culminates in increased α-tubulin degradation by calpains [116]. Recently, HDAC6 up-regulation was proven capable of triggering atrial fibrosis and Cx lateralization in a rat AF model [117].

In PeAF patients, increased markers of DNA damage were positively associated with poly(ADP-ribose) polymerase (PADP) levels and hint an energy-deficient state. In particular, the physiologic cellular process of DNA repair sometimes leads to exaggerated PADP activity and nicotinamide adenine dinucleotide (NAD^+^) depletion, which in turn confers oxidative stress and progressive ATP decline [118].

Additionally, increased production of reactive oxygen species (ROS) is implicated in human AF via both local (atrial cardiomyocyte) and systematic (serum) nicotinamide-adenine dinucleotide phosphate oxidase (NOX) activity [119,120]. Low levels of DNA oxidative stress markers in serum or urine from AF patients have been associated with prolonged arrhythmia-free survival [121,122].

Finally, mitochondrial energy production is critically affected in AF patients since reduced oxidative phosphorylation and increased mitochondrial fragmentation lead to ATP depletion [123,124]. POAF patients are also known to exhibit impaired oxidative phosphorylation capacity pre-operatively [125].

Long noncoding RNAs might be involved in mitochondrial bioenergetics, regulating ATP synthase and CYP450 enzymes [126]. Mitochondrial dysfunction is postulated to induce electrical remodeling via oxidative dysfunction of RyR2 [127].

Adenosine monophosphate-regulated protein kinase (AMPK) activity demonstrates a compensatory increase as a response to AF-induced metabolic stress, restoring calcium homeostasis [128], and it is suggested to be a novel therapeutic target.

Evidently, AF pathogenesis involves overlapping cellular and molecular perturbations that hinder us from distinguishing the cause from the effect (see Appendix A). Since gauging the critical importance of any single mechanism in different clinical AF subtypes is both impractical and unsettled, many therapeutic strategies target multiple mechanisms and seem promising, as discussed in the following section.

## 3. Therapeutic Perspectives

### 3.1. Atrial Selective Ion Channel Inhibitors and Gene Therapy

The limited efficacy of current antiarrhythmic drugs in parallel with their pro-arrhythmic potential and their contraindication in the presence of structural heart diseases dictate the employment of atrial selective anti-AF molecular targets.

A computer-simulated (in silico) model of human AF has recently tested the combination of PVI and I_K,Ach_ blockade against AF development and suggested that this strategy remains highly effective even in cases of severe atrial fibrosis [129].

Niferidil was shown to prolong atrial APD in a porcine model of AF, an effect attributed to I_K,Ach_ and I_K,r_ (delayed rectifier potassium current) inhibition [130]. A sheep model of AF demonstrated that chloroquine exerts anti-AF effects via I_K,Ach_ channel blockade [131]. An equine AF model revealed that XAF-1407, a potent and selective I_K,Ach_ inhibitor, is a safe and effective means of AF cardioversion [132]. This observation is in accordance with Sobota V et al., showing that XAF-1407 demonstrates superior cardioversion efficacy than vernakalant in a goat model of AF [133].

Pentamidine analogue 6 (PA-6) is a synthetic and selective I_K1_ inhibitor capable of restoring sinus rhythm in goats suffering from AF, and it is also known to block I_K1_ current in human atria [134].

The tandem of P domains in a weak inward rectifying K^+^ channel (TWIK)-related acid-sensitive K^+^ channel (TASK-1) is implicated in atrial fibrillation genesis via atrial APD shortening. TASK-1 current inhibition through either siRNA (small interfering RNA) [135] or high-affinity antagonist A293 [136] represent a novel antiarrhythmic strategy. Furthermore, doxapram, a respiratory stimulant and TASK-1 inhibitor, was effective in reducing AF burden in a porcine model of persistent AF [137].

Undeniably, the above-mentioned atrial selective antiarrhythmic substances hold promise in the future pharmacopoeia against AF, yet translational discrepancies must be kept in mind when evaluating novel antiarrhythmic targets. Previous studies have failed to confirm the preclinical antiarrhythmic efficacy of I_K,Ach_ inhibition in human subjects [138]. Additionally, XEN-D0103 (an atrial selective inhibitor of the Kv1.5 channel, which mediates ultra-rapid delayed rectifier current (I_Kur_) [139]) failed to reduce AF burden in PAF subjects [140].

AF gene therapy is still in its infancy, yet promising evidence is constantly accumulating. Following epicardial delivery of HERG-G628S, a dominant-negative mutant of the I_Kr_ channel, porcine atria demonstrated APD prolongation and reduced AF susceptibility [141,142,143,144].

Cx43 gene transfer to porcine atria through epicardial electroporation has been found effective in preventing AF development [145]. Epicardial transfer of Cx40 and Cx43 genes also prevented AF episodes in a swine model of AF via atrial conduction improvement [146].

In a canine model of AF, epicardial delivery of the angiotensin-converting enzyme-2 (ACE2) gene was associated with reduced AF inducibility due to a positive effect on electrophysiological properties of atrial fibroblasts [147].

### 3.2. Drug Repurposing

Drug repurposing has been employed in combating the AF burden and evidently offers good prospects.

Vanoxerine is an oral multi-ion channel blocker (I_Na_, I_Kr_, I_Ca,L_) effective in terminating recent-onset AF, yet the two available randomized placebo-controlled trials (RCTs) reported discordant safety results. In COR-ART, no ventricular proarrhythmia was noticed [148], while RESTORE SR was prematurely terminated due to increased incidence of torsades de pointes ventricular tachyarrhythmia in the vanoxerine arm [149]. A recent pharmacodynamics study highlights that vanoxerine possesses poor atrial selectivity, prolongs ventricular repolarization, and increases intracellular calcium overload risk, thus rendering future investigations unlikely [150].

Antazoline is an anti-histaminic drug that has also been repurposed for AF management due to its multi-ionic effects and has shown effectiveness in AF cardioversion during PVI [151,152]. In addition, a small RCT of 74 paroxysmal AF subjects (AnPAF study) demonstrated that intravenous antazoline is effective in rapid cardioversion of AF [153]. A retrospective case-control study (*N* = 334) suggests that antazoline antiarrhythmic potential is maintained in coronary artery disease patients without safety concerns [154]. CANT study, a retrospective observational study of 450 AF patients treated with pharmacologic cardioversion in the emergency department, showed that antazoline is superior to amiodarone and at least as effective as propafenone [155]. The effectiveness of antazoline in AF cardioversion is also supported by a recent meta-analysis (odds ratio: 24.9; 95% credible interval: 7.4–107.8) [156].

Ivabradine is an inhibitor of I_f_ current, and it is currently approved for the treatment of angina and heart failure. Apart from the sinus node, I_f_ channels are present in the atrioventricular node (AV node), and I_f_ current inhibition can slow AV node conduction in animal studies [157].

Ventricular rate control was achieved after ivabradine administration in a small case series of 6 paroxysmal or persistent AF patients on a maximally tolerated dose of beta-blocker [158]. Wongcharoen W et al. conducted a small RCT (*N* = 32 non-paroxysmal AF subjects) and found that after one month of ivabradine administration, the mean ventricular rate was significantly decreased, as assessed via 24-hour Holter [159].

BRAKE-AF project will compare ivabradine to digoxin in 232 patients with permanent AF and poor rate control despite beta-blocker or calcium channel blocker administration [160]. A meta-analysis of three RCTs (36,577 participants) suggests that ivabradine administration is associated with symptomatic bradycardia and atrial fibrillation (OR: 6.23; CI: 4.2–9.26; *p* < 0.00001 and OR: 1.35; CI: 1.19–1.53; *p* < 0.0001, respectively) [161]. Before putting ivabradine to the test against rate control, these findings necessitate further elucidation.

Dantrolene inhibits sarcoplasmic calcium release in skeletal muscle cells, and it is indicated in malignant hyperthermia. A favorable anti-AF effect has been observed in various preclinical settings such as rat models of heart failure [162], hypertension [163], and autonomic nervous system imbalance [164,165]. A canine model of tachypaced AF further supports dantrolene’s antiarrhythmic potential, and RyR2 channel stabilization is the underlying antiarrhythmic mechanism [166]. RyR2 channels from human atrial cardiomyocytes were also positively affected after in vitro dantrolene treatment [167].

### 3.3. Anti-Fibrotic Agents

Finerenone, a novel mineralocorticoid receptor antagonist, was recently shown to exert antiarrhythmic effects. In particular, the FIDELIO-DKD study evaluated 5,674 patients with chronic kidney disease (eGFR ≥ 25 to <75 mL/min/1.73 m^2^) and type 2 diabetes mellitus; approximately 8% had baseline atrial fibrillation or flutter; on secondary analysis, investigators found that finerenone administration is associated with 29% reduction in new atrial arrhythmic events (HR: 0.71; 95% confidence interval: 0.53–0.94; *p* = 0.016) after a median follow-up of 2.6 years. Of note, Kaplan–Meier curves separation became apparent at 6 months, which implies that structural remodeling reversal might be implicated in finerenone mediated AF prevention [168].

Relaxin is a human hormone belonging to the insulin superfamily and mediates vasodilation, angiogenesis, and hemodynamic changes during pregnancy. Animal studies support an anti-AF potential through fibrosis reversal. In particular, rat models of AF have shown that relaxin administration is associated with TGF-β, metalloproteinase-2, metalloproteinase-9, and collagen I/III downregulation, which in turn enhance atrial conduction velocity by reverting atrial structural remodeling [169,170]. Wnt-signaling was recently shown to mediate relaxin antiarrhythmic effects [171]. Of note, pre-ablation relaxin in serum levels independently predicts radiofrequency ablation outcomes in humans suffering paroxysmal or persistent AF [172]. Serelaxin, a recombinant form of human relaxin, was previously shown to be effective in alleviating dyspnea and reduce all-cause mortality in patients with acute heart failure irrespective of AF status [173]. Whether serelaxin exerts any antiarrhythmic effect, remains to be tested.

As previously discussed, TGF-β/Smad signaling pathway is implicated in atrial fibrosis and represents a therapeutic molecular target. Recently, it was shown that recombinant human MFGE8 (milk fat globule-EGF factor 8) inhibits this pathway and alleviates structural remodeling in atriums of rats [174].

### 3.4. Immunomodulators and AMPK Activators

The inflammatory milieu is involved in both atherosclerosis and AF progression. CANTOS trial suggested that subcutaneous administration of canakinumab at a dose of 150 mg every 3 months reduces the composite endpoint of nonfatal myocardial infarction, nonfatal stroke, or cardiovascular death (hazard ratio: 0.85; 95% CI: 0.74–0.98; *p* = 0.021) [175]. On the contrary, CONVERT-AF a small (*N* = 24) RCT evaluating a single dose of 150 mg canakinumab 60 h after electrical cardioversion for persistent AF failed to reduce 6 month AF recurrence rates (hazard ratio: 0.36; 95% CI: 0.11–1.15; *p* = 0.09) [176].

Colchicine, an inexpensive anti-inflammatory drug with pleiotropic cardioprotective effects [177], is known to reduce POAF incidence [178,179,180], and a recent cost-utility analysis solidifies the cost-effectiveness of this approach [181]. A 3-month scheme of 0.5 mg of colchicine twice daily was previously shown effective in reducing post-ablation AF recurrence after a median follow-up of 15 months [182].

Derailed proteostasis leads to integrated stress response (ISR), which in turn triggers gene expression reprogramming and is implicated in the pathogenesis of many human diseases [183]. In a rat model of post-myocardial infraction AF, inhibition of intracellular stress response (small molecule ISRIB administration) led to AF prevention by halting electroanatomic remodeling, autophagy, and inflammation cascade [184]. In vitro tachypaced canine atrial cardiomyocytes display I_K,Ach_ current enhancement, and calpain inhibition via PD150606 administration led to I_K,Ach_ current suppression and AF prevention [185].

Clinical observational data support that metformin lowers the risk of AF development in patients with diabetes mellitus type 2 [186] and could reduce AF recurrence post-ablation [187]. Preclinical data hint that metformin activates the AMPK pathway and leads to amelioration of mitochondrial dysfunction [188], impaired atrial bioenergetics [189], and connexin dysregulation [190].

Fisetin, a pleiotropic plant polyphenol, was recently tested in a rat AF model. Of note, fisetin treated rodents demonstrated reduced AF susceptibility due to reduced atrial inflammation and fibrosis [191]. AMPK and Smad signaling pathways mediated these effects and imply that simultaneous targeting of various AF molecular perturbations (inflammatory milieu, fibrosis, and bioenergetics) might be an efficacious strategy.

### 3.5. Neuromodulation

Autonomic neuromodulation is an evolving and promising AF therapeutic approach. Botulinum toxin injection into epicardial fat pads was shown effective in reducing post-operative AF episodes during a 3-year follow-up. Temporary interruption of intrinsic cardiac ANS overactivity restores autonomic imbalance and breaks the vicious cycle of AF begetting AF [88]. Interestingly, intermittent low level transcutaneous (ear tragus) vagal stimulation lowers AF burden in patients with paroxysmal AF at 6 months [90]. Again, electroanatomical remodeling reversal and anti-inflammatory effects are seemingly involved in this neuromodulatory strategy.

Furthermore, adiponectin administration into ganglionated plexi in a tachypached canine AF model was associated with AF suppression [192]. This study underscores that the crosstalk between EAT and ANS is a valid therapeutic target to AF and merits further investigation.

Indirect cardiac neuromodulation can also be achieved via renal sympathetic denervation (RDN). AFFORD study showed that patients with AF and hypertension undergoing RDN benefited in terms of blood pressure control and AF burden reduction [193]. A small (*N* = 432) meta-analysis by Atti V et al. demonstrated that RDN plus PVI is superior to PVI alone in reducing post-ablation arrhythmia recurrence in patients with AF (paroxysmal or persistent) and concomitant hypertension (RR: 0.58; 95% CI: 0.47–0.72; *p* < 0.00001) [194].

The central sympatholytic agent moxonidine was previously shown effective in reducing AF burden in patients with paroxysmal AF [195] as well as 12-month arrhythmia recurrence rates following PVI for paroxysmal AF [196].

Multipulse Therapy (MPT) constitutes an intracardiac sequence of low-voltage, low-energy stimulation pulses aiming to terminate AF episodes. The first human feasibility study showed 71% efficacy in terminating AF and paves the way for further investigations, with the ultimate goal being MPT implementation in cardiac devices (implantable cardioverter defibrillators and resynchronization cardiac therapies) [197].

## 4. Conclusions

Inflammatory milieu and metabolic stress (mitochondrial dysfunction and derailed proteostasis) are interlinked with the classic electroanatomic remodeling in human AF. The molecular mediators of these pathways should be targeted in order to combat AF pathogenesis. Hopefully, novel therapeutic approaches are underway, and clinical data are much awaited.

## Figures and Tables

**Figure A1:**
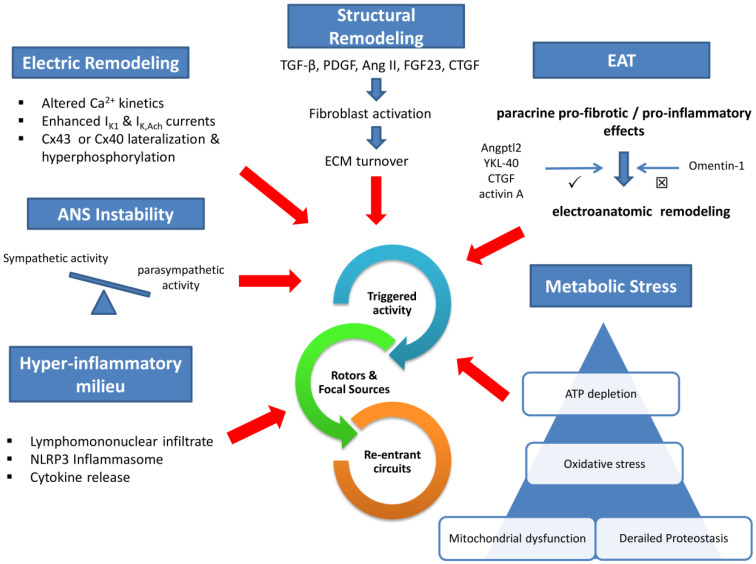
Central Illustration Legend: Six overlapping and interlinked (not shown) pathways are implicated in atrial fibrillation pathophysiology: electric remodeling, structural remodeling, autonomic instability, hyperinflammatory milieu, metabolic stress, and epicardial adipose tissue paracrine effects. The common endpoint is the initiation and maintenance of arrhythmic events. I_K1_—inwardly rectifying potassium current; I_K,Ach_—acetylcholine-activated potassium current; ANS—autonomic nervous system; Cx—connexin; TGF-β—tissue growth factor β; PDGF—platelet-derived growth factor; AngII—angiotensin II; FGF23—fibroblast growth factor 23; CTGF—connective tissue growth factor; ECM—extracellular matrix; EAT—epicardial adipose tissue; Angptl2—angiopoietin-like protein 2.
